# Impact of statin use on survival and adverse events in patients with cancer receiving radiotherapy: a systematic review and meta-analysis

**DOI:** 10.1186/s12885-025-15038-3

**Published:** 2025-10-29

**Authors:** Hala Shokr, Wan-Chuen Liao, Corinne Faivre-Finn, Clare Dempsey, Kaye Janine Williams, Li-Chia Chen

**Affiliations:** 1 Division of Pharmacy and Optometry, School of Health Sciences, Faculty of Biology, Medicine and Health, The University of Manchester, Oxford Road, Manchester, M13 9PT UK; 2 School of Dentistry, College of Medicine, National Taiwan University, Taipei, 100229 Taiwan; 3 Division of Cancer Sciences, Faculty of Biology, Medicine and Health, The University of Manchester, Manchester, M13 9PT UK; 4 The Christie NHS Foundation Trust, Manchester, M20 4BX UK

**Keywords:** Radiotherapy, Polypharmacy, Radiotherapy-drug interaction, Statin, Cancer, Survival outcome, And adverse effects

## Abstract

**Background:**

Given limited and conflicting data, this systematic review and meta-analysis investigate the impacts of statin use on survival outcomes and adverse events in patients with cancer receiving radiotherapy.

**Methods:**

A comprehensive search of MEDLINE, EMBASE, Web of Science, Scopus, and PubMed (January 2000 to June 2024) included studies on adults (≥ 18 years) with histologically confirmed cancer receiving oral statins during radiotherapy. Overall survival (OS) rates and radiotherapy-related adverse effects were compared between statin users and non-users using odds ratios (ORs) and 95% confidence intervals (95%CIs). Meta-regression explored the effects of cancer type and statin intensity on OS rates, reported as coefficients (β) and 95%CI.

**Results:**

Of 21 studies (19 cohort studies and two randomized trials), OS rates did not significantly differ between statin users and non-users (OR: 1.29; 95%CI: 0.99, 1.69) or by statin intensity (β: 0.20; 95%CI: -1.22, 1.62; *p* = 0.60), but significantly by cancer types (β: -0.29; 95%CI: -0.45, -0.13; *p* < 0.01). Statin use was associated with improved survival in oesophageal squamous cell carcinoma (SCC), head and neck SCC, glioblastoma, and prostate cancer, but with reduced survival in non-small cell lung cancer (NSCLC) and brain metastases. Statin users had a higher risk of major adverse cardiac events (OR: 2.22; 95%CI: 1.38, 3.59) in NSCLC and ≥ grade 2 mucositis (OR: 26.00; 95%CI: 4.09, 165.10) in head and neck squamous cell carcinoma but lower risks of ischemic stroke (OR: 0.80; 95%CI: 0.67, 0.95) in nasopharyngeal carcinoma and rectal toxicity (OR: 0.45; 95%CI: 0.23, 0.88) in prostate cancer.

**Conclusions:**

Survival outcomes did not significantly differ by statin use or intensity but varied by cancer type. Statin users had lower risks of ischemic stroke and rectal toxicity. Further studies are needed to control for confounding biases.

**Trial registration:**

PROSPERO registration CRD42023487336.

**Supplementary Information:**

The online version contains supplementary material available at 10.1186/s12885-025-15038-3.

## Background

Polypharmacy is prevalent among the elderly population as well as in patients with cancer [1, 2]. Balancing the risks and benefits of continuing multiple concurrent medications during cancer radiotherapy is imperative in clinical oncology. Still, a lack of data remains to inform clinical decisions [3]. Statins, cholesterol-lowering agents, are commonly prescribed long-term to middle-aged patients with hypercholesterolemia for the prevention of cardiovascular and coronary heart diseases due to their pleiotropic anti-inflammatory, antioxidant, and anti-fibrotic effects [4, 5]. In the United Kingdom, 24.6% of middle-aged adults (aged 40 and above) reported using statins in 2018 [6]. Statins are also highly prevalent in patients with cancers receiving radiotherapy [7].

Statins have diverse cellular effects, including regulating cell proliferation, differentiation, and survival [8, 9]. They inhibit 3-hydroxy-3-methylglutaryl CoA reductase, the rate-limiting enzyme in the mevalonate pathway, leading to decreased cholesterol synthesis and downstream isoprenoid intermediates [10]. These intermediates are crucial for the post-translational modification of proteins, which are involved in cell proliferation, survival, and migration. Statins can also arrest cells in the late G1 phase of the cell cycle, disrupting synchronisation during the radiosensitive phase and potentially reducing radiation resistance [11].

Some review articles have addressed the ability of statins to inhibit the proliferation and induce apoptosis of tumour cells, suggesting their broader therapeutic potential as an adjuvant to cancer treatment [8, 12]. Radiotherapy may benefit from statins by potentially mitigating typical tissue damage through reductions in pro-inflammatory and pro-fibrotic cytokines, as well as moderating the DNA damage response triggered by ionising radiation [13]. However, conflicting and controversial clinical results regarding radiotherapy still exist.

Most prior research investigating the associations between concurrent statin use and various cancers in patients receiving radiotherapy was retrospective cohort observational studies. Statin use has been suggested to improve cancer outcomes in patients with head and neck cancer [7, 14], oesophageal squamous cell carcinoma (SCC) [15], and pelvic malignancies [16]. Conversely, other research has shown no effect of statin use on glioblastoma [17] and brain metastases [18]. In patients with prostate cancer and non-small cell lung cancer (NSCLC) receiving radiotherapy, both beneficial [19–26] and no effects [15, 27–30] of statin use have been reported.

In addition, recent retrospective studies revealed a dose-response relationship between statin intensity and survival outcomes in patients with NSCLC (*n* = 478) and oesophageal SCC (*n* = 420) undergoing radiotherapy or concurrent chemoradiotherapy [26, 31]. Higher cumulative daily doses or intensity of statin use were associated with reduced mortality and better overall survival [26, 31]. Conversely, a retrospective cohort study of patients with prostate cancer (*n* = 774) receiving external beam radiation therapy found no clear dose-response relationship for daily statin dose or duration of use [29]. Variations in follow-up periods and actual drug durations likely contribute to these differences.

Conflict ing results from prospective trials and retrospective studies highlight the need to integrate current evidence for oncology practice recommendations. Assessing and synthesizing diverse findings comprehensively is crucial to inform evidence-based clinical decisions on combining statins with radiotherapy in cancer treatment. Therefore, this systematic review and meta-analysis aimed to clarify the impact of concurrent statin use on survival outcomes and radiotherapy-related adverse events in patients undergoing radiotherapy by synthesising existing literature.

## Methods

This systematic review and meta-analysis adhered to the Preferred Reporting Items for Systematic Reviews and Meta-Analyses (PRISMA) statement guidelines (Appendix 1) [32]. The protocol was registered at PROSPERO (no. CRD42023487336).

### Selection criteria

The inclusion and exclusion criteria of this study (Table 1) are summarised as follows.

**Table 1:** Inclusion and exclusion criteria of this study

Component	Inclusion criteria	Exclusion criteria
Population and conditions	• Patients aged 18 years and above. • Patients diagnosed with histologically confirmed cancers (newly diagnosed or recurrent) are scheduled to receive radiotherapy.	• Patients include paediatrics, children, adolescents, neonates, and infants. • Studies involved mixed-age groups. • Neoadjuvant radiotherapy or diagnostic radiology (e.g., X-rays, magnetic resonance images). • Patients with cancer types are not amenable to radiotherapy.
Intervention and comparator	Oral administration of statin, either alone or in combination with other drugs, such as chemotherapy.	• Non-concurrent use of statin and radiotherapy (not during the radiotherapy cycles).
Outcome	• Survival outcomes included overall survival and other related results. • Adverse events occurred during or right after the radiotherapy.	• Radiation-related toxicity occurred before the administration of statin.
Study type	Human studies	Animal or in vitro studies
Language	English	Other languages without English translation
Publication	Full-text article on prospective or retrospective cohort study, cross-sectional study, and clinical trial.	Case-control study, case series, case report, systematic review, meta-analysis, conference abstract, abstract without full article, editorial, letter to editors, commentary, and grey literature.

### Types of studies

Original articles of prospective and retrospective cohort studies, cross-sectional studies, and clinical trials were included. Case-control studies, case series, case reports, systematic reviews, meta-analyses, conference abstracts, editorials, letters to editors, commentary, and grey literature were excluded (Table 2).

**Table 2:** Characteristics of included studies

Author, year, country	Cancer	Types of radiation	Radiation dose (Gy)	Number of patients			Age of patients (year)	Outcome category
				Total	Statin users	Non-users		
Moyad, 2006, US [30]	Localized prostate cancer	Brachytherapy	NA	938	191	747	Mean ± SD: 66.1 ± 7.2	Survival outcomes
Soto, 2009, US [18]	Localized prostate cancer	Definitive RT	Median (range): 75.8 (45, 153)	968	220	748	Mean ± SD: 68.2 ± 7.3	Survival outcomes
Gutt, 2010, US [20]	Prostate cancer	EBRT and/or brachytherapy	Median (range): 72 (NA)	691	189	502	Median (range): statin user: 69 (42, 83); non-user: 68 (44, 83)	Survival outcomes
Kollmeier, 2011, US [22]	Prostate cancer	RT	Median (range): 81 (75.6, 86.4)	1681	382	1299	NA	Survival outcomes
Alizadeh, 2012, Canada [19]	Prostate cancer	EBRT or brachytherapy	NA	381	172	209	Mean ± SD: statin user: 66.0 ± 6.0; non-user: 65.9 ± 7.4	Survival outcomes
Wedlake, 2012, UK [16]	Pelvic malignancies	Radical pelvic RT	Median (range): statin user: 64 (36, 74); non-user: 55.8 (20, 74)	237	38	199	Median (range): statin user: 73.5 (59, 86); non-user: 67 (29, 88)	RT-related side effects
Chao, 2013, US [29]	Prostate cancer	EBRT	NA	774	401	373	Mean ± SD: 68.4 ± 7.0	Survival outcomes
Caon, 2014, Canada [28]	Localized prostate cancer	EBRT	Median (range): 70 (52.50, 78)	2934	506	2428	Mean (range): 70.3 (45, 88)	Survival outcomes
Cuaron, 2015, US [15]	Prostate cancer	Brachytherapy	Median: patients received either LDR (144) or HDR (38) monotherapy or LDR (110) or HDR (19.5) in combination with supplemental EBRT (50.4)	754	273	481	NA	Survival outcomes
Oh, 2015, US [24]	Prostate cancer	Brachytherapy	Brachytherapy: 145 or 110; EBRT: range: 22, 46	247	174	73	Median (range): 62 (45.6, 81.94)	Survival outcomes
El-Hamamsy, 2016, Egypt [33]	Brain metastases	Whole-brain RT	Median (range): 30 (NA)	30	15	15	Mean ± SD: 54.4 ± 11.1	Survival outcomes
Liu, 2017, US [23]	Prostate cancer	RT	Median (range): 2000–2005: 75.6 (NA); 2009–2012: 80.3 (NA)	381	146	235	Mean ± SD: 74.4 ± 6.0	Survival outcomes
Palumbo, 2017, Italy [25]	Prostate cancer	Hypofractionated intensity-modulated RT	Median (range): 74.25 (NA)	195	55	140	Median (range): 74 (57, 85)	RT-related side effects
Boulet, 2019, Canada [7]	Thorax, head and neck cancer	RT	NA	5718	4166	1552	Mean ± SD: 75 ± 6.1	Survival outcomes RT-related side effects
Cadeddu, 2020, Spain [27]	High-risk prostate cancer	RT	Range: 72, 76	447	175	272	Median (range): 70 (46, 83)	Survival outcomes RT-related side effects
Altwairgi, 2021, Saudi Arabia [17]	Glioblastoma	RT	Median (range): 12 (NA)	388	36	352	Median (range): statin user: 52 (20, 69); historical control: 56 (19, 70); control trial: 47 (18, 81)	Survival outcomes
Atkins, 2021, US [34]	Locally advanced NSCLC	Thoracic RT	Median (IQR): statin user: 64 (56, 66), non-user: 64 (54, 66)	748	305	443	Median (IQR): statin user: 67 (61, 75); non-user: 62 (55, 71)	Survival outcomes RT-related side effects
Chen, 2023, Taiwan [31]	Oesophageal SCC	CRT	Total dose: 50.4	420	140	280	Mean ± SD: statin user: 64.23 ± 11.93; non-user: 64.53 ± 13.27	Survival outcomes
Walls, 2023, UK [26]	NSCLC	RT	NA	478	283	195	Median (IQR): 70 (64, 76)	Survival outcomes RT-related side effects
Lin, 2024, Taiwan [14]	Advanced nasopharyngeal carcinoma	RT	Range: 70, 70.2	5022	2515	2507	Median (IQR): statin user: 51.30 (46.07, 59.01); non-user: 51.11 (43.91, 59.15)	RT-related side effects
Sharifian, 2024, Iran [33]	Locally advanced head and neck SCC	CRT	Total dose: 70	35	18	17	Mean: statin user: 57.9; non-user: 57.2	Survival outcomes RT-related side effects

*US* United States, *UK* United Kingdom, *NSCLC* non-small cell lung cancer, *SCC* squamous cell carcinoma, *RT* radiotherapy, *EBRT* external beam radiation therapy, *CRT* chemoradiotherapy, *NA* not available, *LDR* low dose rate, *HDR* high dose rate, *IQR* interquartile range, *SD* standard deviation

### Types of participants

Studies enrolled participants aged 18 and above with histologically confirmed cancer undergoing various radiotherapy doses and regimens were included. Studies involving individuals under 18 years or mixed-age groups, those undergoing neoadjuvant radiotherapy or diagnostic radiology, and those with cancer not indicated for radiotherapy were excluded.

### Types of interventions

Studies of patients receiving oral statins alone or combined with other drugs (e.g., chemotherapy) during radiotherapy were included. Studies of patients not using statins concurrently during radiotherapy cycles were excluded.

### Types of outcome measures

Included were studies measuring survival outcomes (e.g., overall survival rate or survival time) and radiotherapy-related side effects occurring during or immediately after radiotherapy. Excluded were studies that reported only radiation-related toxicity occurring before statin administration.

### Data sources and search strategies

A comprehensive search of electronic databases, including MEDLINE, EMBASE, Web of Science, Scopus, and PubMed, was conducted from January 2000 to June 2024. The search was restricted to studies published from January 2000 onward, focusing on research conducted in the context of established statin use, following landmark trials such as the Heart Protection Study [35], which significantly influenced clinical practice starting in 2002. This approach aimed to capture literature reflecting current prescribing patterns and clinical relevance. The initial exploration revealed pertinent literature published after 2000, prompting the commencement of the review in July 2024. This was achieved by applying structured search strategies (Appendix 2), which incorporated controlled vocabulary and keywords aligned with predefined inclusion and exclusion criteria (Table 1). Additionally, the search was restricted to English-language publications and human studies.

### Study selection

Two reviewers (WCL and HS) independently screened titles and abstracts of articles retrieved from the electronic database search using a pre-designed form and categorised studies as “included,” “further check,” or “excluded.” Prior to the screening, a calibration exercise was conducted where both reviewers independently screened a sample of records. Consistency between reviewers was evaluated using the intraclass correlation coefficient (two-way mixed effect model with absolute agreement) [36]. Disagreements were resolved through discussion between reviewers, and a third reviewer (LCC) was consulted if needed to reach a consensus. Potentially eligible articles underwent further independent review by both reviewers (WCL and HS) to finalise inclusion decisions.

### Data extraction and management

Two reviewers (WCL and HS) independently used a standardized electronic data extraction form to extract study data. Disagreements were resolved by a third reviewer (LCC). Extracted information included study details such as title, lead author, country, publication year, study design, setting, targeted population (disease and cancer stages), intervention (type and dosage of statin), comparison, outcome measures, and follow-up period. Study results were retrieved, including survival outcomes and adverse events during or immediately after radiotherapy. If raw data were unavailable, mean (with standard deviation) or median (range) values were extracted. Statin intensity was categorised based on the guidelines into low, medium, and high [37, 38].

### Risk of bias assessment

All included studies underwent quality assessment using the Cochrane Risk of Bias Assessment Tool (RoB 2) [39] for randomized controlled trials and the Risk of Bias in Non-randomized Studies of Interventions tool (ROBINS-I) [40] for non-randomized studies. Studies were classified as having a low risk of bias, some concerns, or high risk of bias according to RoB 2 and low, moderate, serious, or critical risk of bias based on ROBINS-I.

### Data analysis

Survival outcomes and radiotherapy-related side effects were compared between statin users and non-users. If studies reported survival outcomes at various time points, priority was given to the closest censoring year to the five-year mark, as the five-year survival rate is widely acknowledged as a critical measure of cancer care quality and long-term outcomes [41].

Overall survival rate, as well as the progression-free, cause-specific, and distant metastasis-free survival rates, were synthesized using a random-effects model (Der-Simonian and Laird method [42]), and the pooled effect size was presented as odds ratio (OR) with 95% confidence interval (95%CI). Heterogeneity was assessed using the *I*^*2*^ test (%). Meta-regression was used to analyse factors (the type of cancers and intensity of statins) associated with effect size, presenting the results as coefficient (β) and 95%CI.

The median survival times were reported by subtracting between statin users and non-users to demonstrate the difference. The effect size (OR and 95%CI) of radiotherapy-related side effect rate was reported for different types of cancers. STATA (Release 14, College Station, TX: StataCorp LLC) was used for meta-analysis and meta-regression, with statistical significance set at *p* < 0.05.

## Results

### Selection of study

**Fig. 1:**
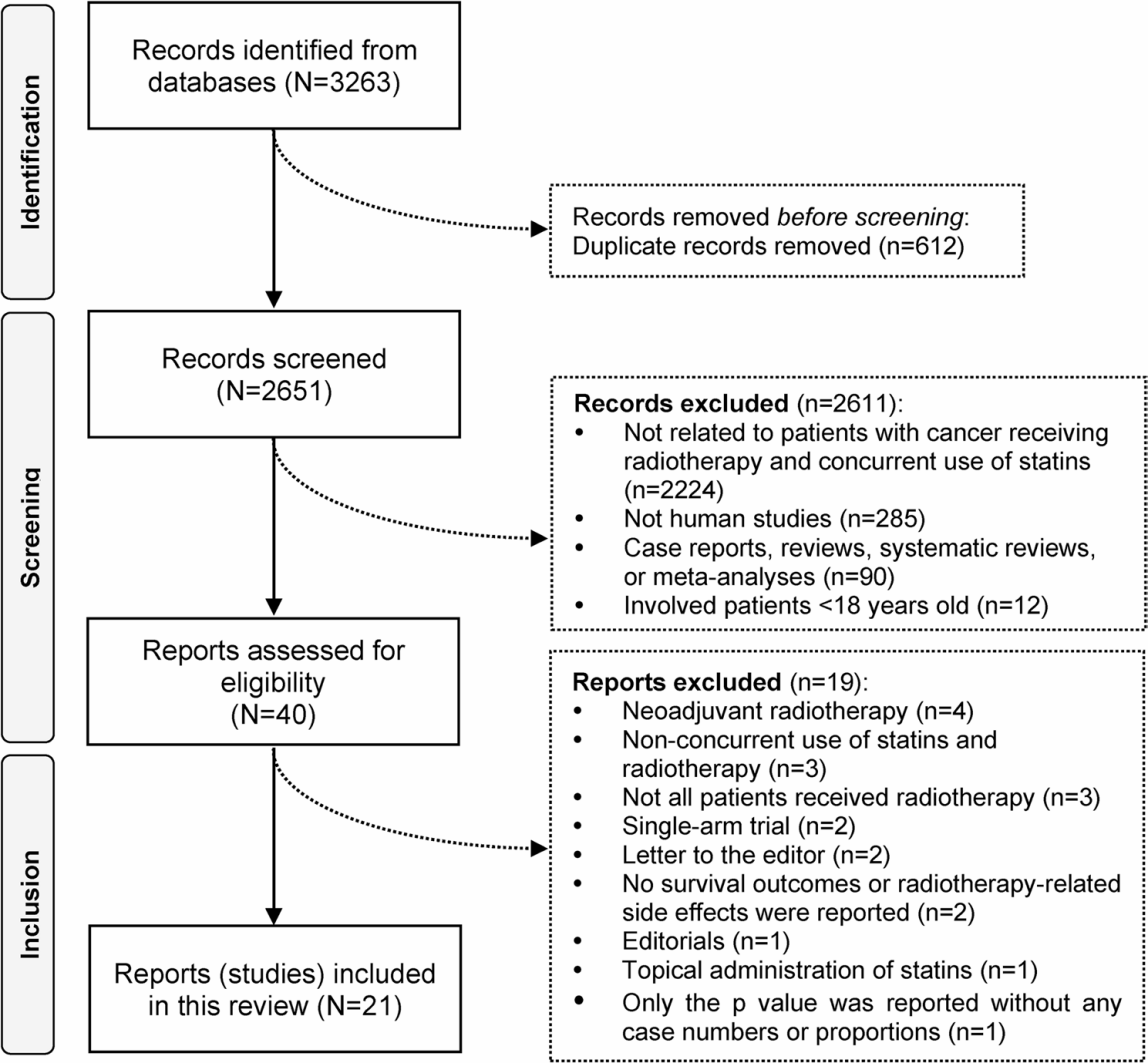
Selection of studies

Of the 3263 records identified from electronic database searches, 40 studies were assessed for eligibility after removing duplicates (*n* = 612) and irrelevant records (*n* = 2611), such as those unrelated to patients with cancer receiving radiotherapy and concurrent use of statins (*n* = 2224), non-human studies (*n* = 285), case reports, reviews, systematic reviews, or meta-analyses (*n* = 90), and studies involving patients under 18 years old (*n* = 12). During the full-text screening, 19 studies were excluded, leaving 21 studies (23467 patients) for analysis (Fig. 1). The intraclass correlation coefficient was 0.961 (95%CI: 0.957, 0.965) between the two reviewers, indicating a good consistency.

### Characteristics of included studies

Most of the 21 included studies (19 cohort studies [7, 14–20, 22–31, 41] and two randomised trials [33, 43]) targeted patients with prostate cancer (*n* = 12) [15, 18–20, 22–25, 27–30], followed by NSCLC (*n* = 2) [26, 34], thorax, head, and neck cancer (*n* = 2) [7, 43], pelvic malignancies (*n* = 1) [16], brain metastases (*n* = 1) [33], glioblastoma (*n* = 1) [17], oesophageal SCC (*n* = 1) [31], and nasopharyngeal carcinoma (*n* = 1) [14]. Overall, 10,400 statin users and 13,067 non-users were included in this review.

Four studies provided information on statin dosage and intensity, with two focusing on high-intensity statins [17, 33], one using moderate-intensity lovastatin [43], and one covering low, medium, and high intensities [26]. The most frequently reported survival outcome is the overall survival rate (*n* = 11) [15, 17, 20, 26–28, 30, 31, 33, 34, 43].

The radiotherapy-related adverse events were cancer-specific and reported in patients with prostate cancer (*n* = 2) [25, 27], NSCLC (*n* = 2) [26, 34], thorax, head, and neck cancer (*n* = 2) [7, 43], nasopharyngeal carcinoma (*n* = 1) [14], and pelvic malignancies (*n* = 1) [16].

### Quality assessment

According to RoB 2, two included randomised trials showed a high risk of bias. One was due to the heterogeneity of primary tumour origin, limited follow-up, and the severe cognitive impairment of the patients, which would affect the accuracy of self-rated quality-of-life assessments [33]. The other was missing outcome data in a small sample size trial [43] (Appendix 3).

Among the 19 cohort studies, nine had serious bias [7, 15–17, 19, 22, 26, 29, 30].

and ten had moderate bias [14, 18, 20, 23–25, 27, 28, 31, 34] (Appendix 4). Serious biases included neglecting to report radiation dose (*n* = 5) [7, 19, 26, 29, 30], cancer stage (*n* = 3) [7, 16, 17], follow-up period (*n* = 2) [16, 19], and population age (*n* = 2) [15, 22]. One study had a serious bias in outcome measurement when comparing prospectively collected data with retrospective statistics [17]. Confounding bias existed in statin administration, cancer stage, and patients’ comorbidities.

### Survival outcomes

The overall survival rate did not significantly differ between statin users and non-users (OR: 1.29; 95%CI: 0.99, 1.69; *I*^*2*^ = 69.9%) in the pooled results of 11 studies. Statin users had a significantly better progression-free survival rate (OR: 1.52; 95%CI: 1.12, 2.07; *I*^*2*^ < 0.1%) based on three studies and a distant metastases-free survival rate (OR: 1.73; 95%CI: 1.09, 2.75; *I*^*2*^ < 0.1%) according to two studies. Although not significantly different, statin users had a slightly better cause-specific (death from prostate cancer) survival rate, pooled from two studies, with a wide 95%CI (OR: 4.60; 95%CI: 1.00, 21.20; *I*^*2*^ = 16.2%) (Table 3).

**Table 3:**
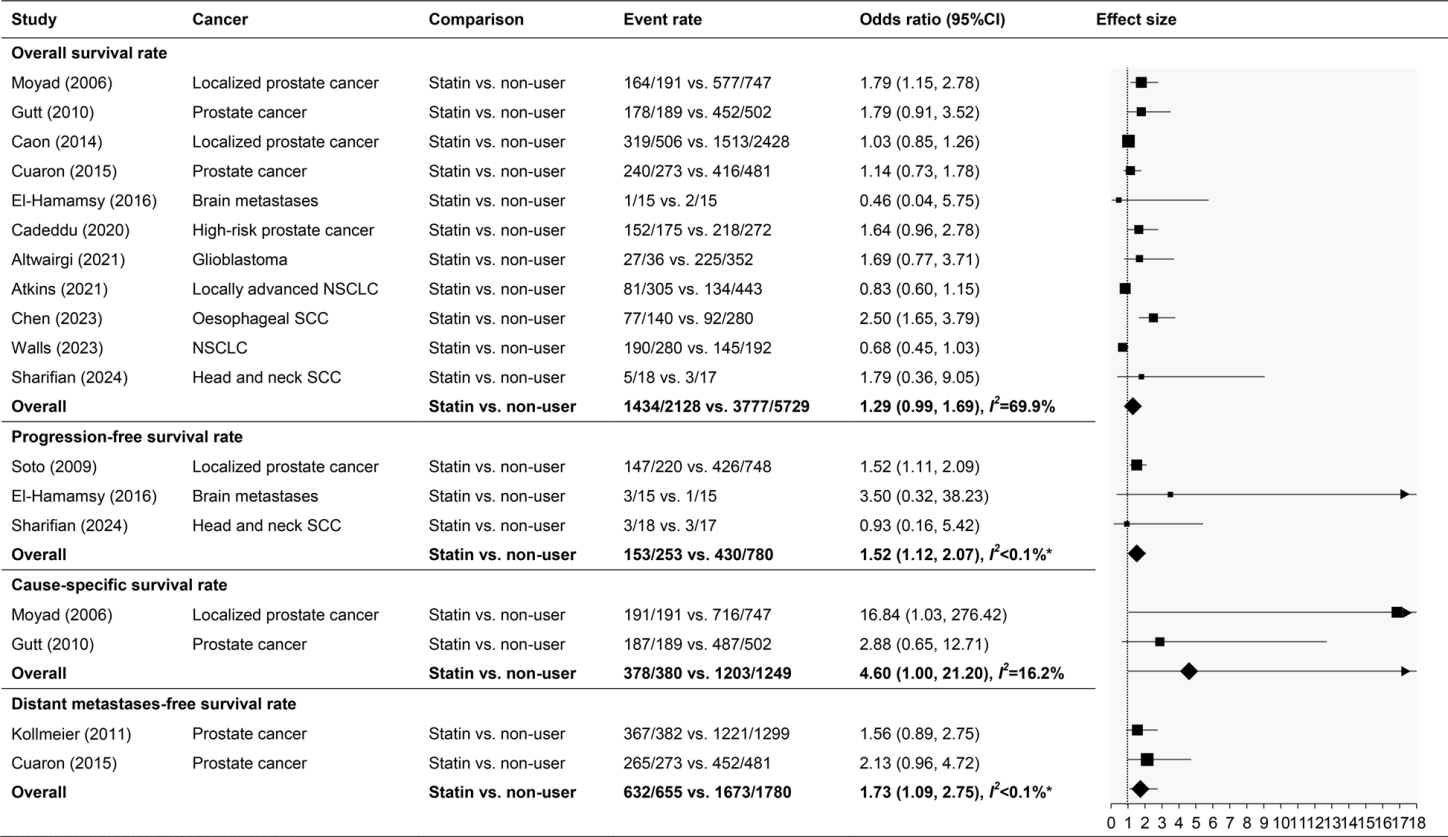
Survival outcome of overall, progression-free, cause-specific, and distant metastases-free survival rate

*NSCLC* non-small cell lung cancer, *SCC* squamous cell carcinoma, *CI* confidence interval

*Significant difference

**Fig. 2:**
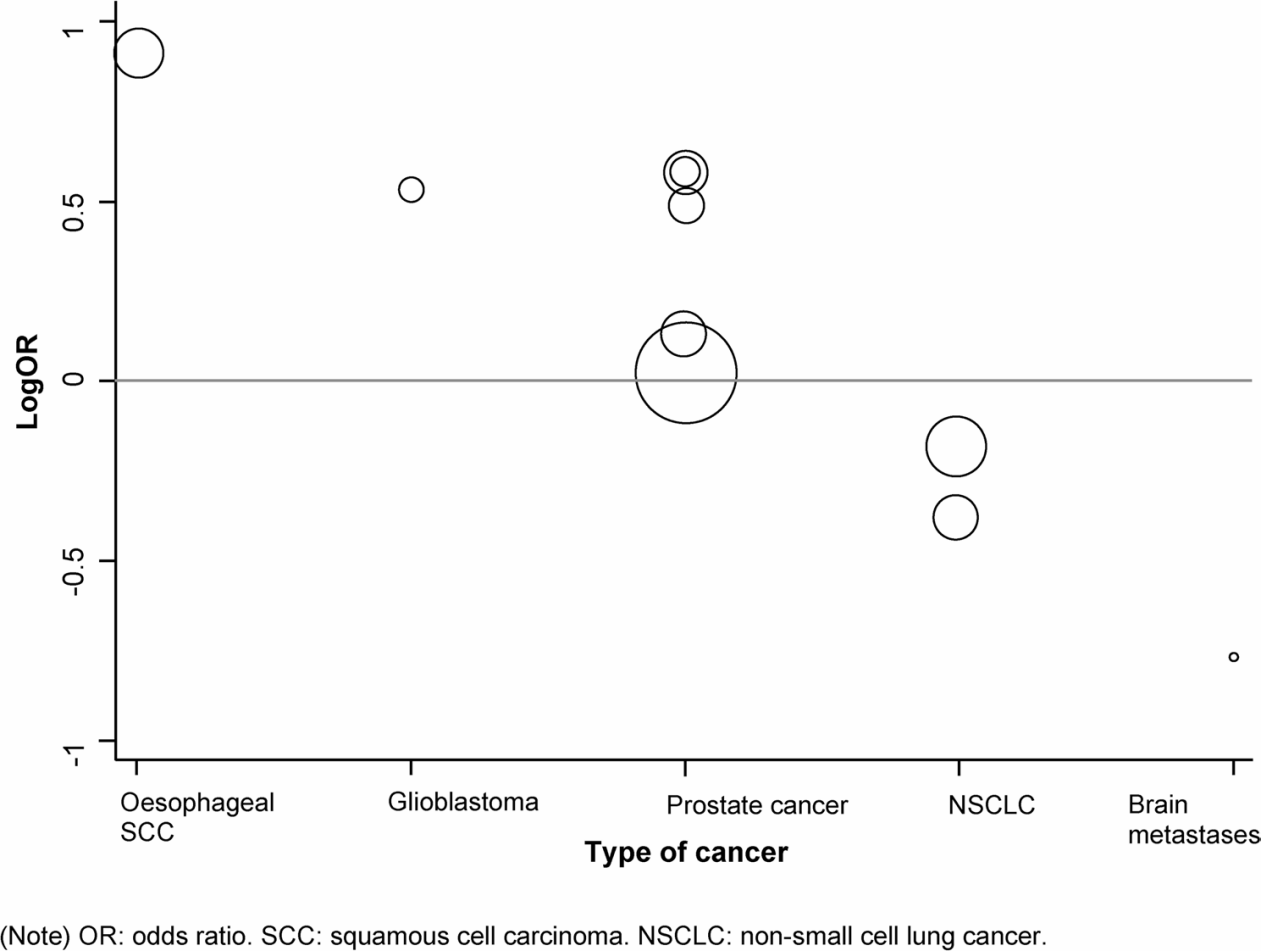
Meta-regression of log odds ratios of overall survival rate and type of cancer

Meta-regression analysis revealed that the type of cancer significantly affects the overall survival rate (β: −0.29; 95%CI: −0.45, −0.13; *p* < 0.01). Statin users had better survival in oesophageal SCC, head and neck SCC, glioblastoma, and prostate cancer, but worse in NSCLC and brain metastases (Fig. 2). Statins did not significantly affect the overall survival rate (β: 0.20; 95%CI: −1.22, 1.62; *p* = 0.60). 

Statin users showed significantly better outcomes in several measures: prostate-specific antigen level > 20 ng/mL rate (OR: 0.29; 95%CI: 0.08, 0.83) [19], prostate cancer-specific survival rate (OR: 1.53; 95%CI: 1.03, 2.28) [28], the cumulative biochemical failure rate in prostate cancer (OR: 0.18; 95%CI: 0.07, 0.50) [24], freedom from biochemical failure in prostate cancer (OR: 4.16; 95%CI: 1.31, 13.18) [24], all-cause mortality in oesophageal SCC (OR: 0.33; 95%CI: 0.21, 0.53) [31], oesophageal SCC-specific survival rate (OR: 2.25; 95%CI: 1.49, 3.40) [31], and oesophageal SCC-specific mortality (OR: 0.45; 95%CI: 0.30, 0.68) [31] compared to non-users (Table 4). Notably, each measure was reported by a single study.

**Table 4:** Other survival outcomes reported by only one study in each indicator

Survival outcome	Study	Comparison	Event rate	Odds ratio (95%CI)
Prostate cancer				
Biochemical progression-free survival rate	Moyad (2006)	Statin vs. non-user	188/191 vs. 711/747	3.17 (0.97, 10.42)
Freedom from distant metastases	Gutt (2010)	Statin vs. non-user	185/189 vs. 482/502	1.92 (0.65, 5.69)
Prostate-specific antigen level > 20 ng/mL rate	Alizadeh (2012)	Statin vs. non-user	NA/172 vs. NA/209 ^§^	0.29 (0.08, 0.83) *
Prostate cancer recurrence rate	Chao (2013)	Statin vs. non-user	81/401 vs. 64/373	1.22 (0.85, 1.76)
Prostate cancer-specific survival rate	Caon (2014)	Statin vs. non-user	476/506 vs. 2214/2428	1.53 (1.03, 2.28) *
Cumulative biochemical failure rate	Oh (2015)	Statin vs. non-user	6/174 vs. 12/73	0.18 (0.07, 0.50) *
Freedom from biochemical failure	Oh (2015)	Statin vs. non-user	169/174 vs. 65/73	4.16 (1.31, 13.18) *
Prostate-specific antigen relapse-free survival rate	Cuaron (2015)	Statin vs. non-user	231/273 vs. 424/481	0.74 (0.48, 1.14)
Biochemical failure-free survival rate	Cadeddu (2020)	Statin vs. non-user	137/175 vs. 221/272	0.83 (0.52, 1.33)
Disease-specific survival rate	Cadeddu (2020)	Statin vs. non-user	172/175 vs. 265/272	1.51 (0.39, 5.94)
Distant failure-free survival rate	Cadeddu (2020)	Statin vs. non-user	158/175 vs. 239/272	1.28 (0.69, 2.38)
Thorax, head and neck cancers				
Myocardial infarction/stroke/death rate	Boulet (2019)	Statin vs. non-user	376/4166 vs. 160/1552	0.86 (0.71, 1.05)
Stroke rate	Boulet (2019)	Statin vs. non-user	110/4166 vs. 56/1552	0.72 (0.52, 1.00)
Oesophageal SCC				
All-cause mortality rate	Chen (2023)	Statin vs. non-user	87/140 vs. 233/280	0.33 (0.21, 0.53) *
Oesophageal SCC-specific survival rate	Chen (2023)	Statin vs. non-user	82/140 vs. 108/280	2.25 (1.49, 3.40) *
Oesophageal SCC-specific mortality rate	Chen (2023)	Statin vs. non-user	66/140 vs. 186/280	0.45 (0.30, 0.68) *

*SCC* squamous cell carcinoma, *NA* not available, *CI* confidence interval

^§^The case number was not provided, but the odds ratio and 95% confidence interval were reported in the included study

*Significant difference

In statin users with brain metastases [33] and head and neck SCC [43], median overall survival and progression-free survival times were longer by less than a month and five months, respectively, compared to non-users. Conversely, in patients with glioblastoma, statin users had a longer median overall survival but shorter median progression-free survival time [17]. Statin users with NSCLC had a median locoregional control of 10.8 months shorter and a median distant control of 17.8 months shorter than non-users [26] (Table 5). Additionally, the mean prostate-specific antigen level was lower in statin users with prostate cancer than in non-users [23].

**Table 5:** Survival outcomes of median survival time

Study	Cancer	Comparison	Number of patients	Median time (months)	Median time differences between exposed and non-exposed groups (months)
Overall survival time					
El-Hamamsy (2016) [33]	Brain metastases	Statin vs. non-user	15 vs. 15	3.4 vs. 3	0.4
Altwairgi (2021) [17]	Glioblastoma	Statin vs. non-user	36 vs. 352	19.9 vs. 19.6	0.3
Sharifian (2024) [43]	Head and neck SCC	Statin vs. non-user	18 vs. 17	22 vs. 17	5
Progression-free survival time					
El-Hamamsy (2016) [33]	Brain metastases	Statin vs. non-user	15 vs. 15	1.6 vs. 1.47	0.13
Altwairgi (2021) [17]	Glioblastoma	Statin vs. non-user	36 vs. 352	7.6 vs. 7.8	−0.2
Sharifian (2024) [43]	Head and neck SCC	Statin vs. non-user	18 vs. 17	20 vs. 15	5
Locoregional control					
Walls (2023) [26]	NSCLC	Statin vs. non-user	283 vs. 195	29.7 vs. 40.5	−10.8
Distant control					
Walls (2023) [26]	NSCLC	Statin vs. non-user	283 vs. 195	34.1 vs. 51.9	−17.8

*SCC* squamous cell carcinoma, *NSCLC* non-small cell lung cancer

### Radiotherapy-related adverse events

Statin users had a significantly higher risk of major adverse cardiac events in patients with NSCLC (OR: 2.22; 95%CI: 1.38, 3.59) [34] and ≥ grade 2 mucositis (OR: 26.00; 95%CI: 4.09, 165.1) in head and neck SCC, but a lower risk with ischemic stroke (OR: 0.80; 95%CI: 0.67, 0.95) in patients with nasopharyngeal carcinoma [14] and rectal toxicity (OR: 0.45; 95%CI: 0.23, 0.88) in those with prostate cancer [25] compared to non-users (Table 6). Additionally, statin users with pelvic malignancies had higher inflammatory bowel disease questionnaire-bowel scores, indicating fewer symptoms [16].

**Table 6:** Radiotherapy-related adverse events

Adverse event	Study	Comparison	Follow-up (months)	Event rate	Odds ratio (95%CI)
NSCLC					
≥ 1 major adverse cardiac events rate	Atkins (2021)	Statin vs. non-user	Median (IQR): 20.4 (8.4, 45.0)	45/305 vs. 32/443	2.22 (1.38, 3.59) *
Cardiac events rate	Walls (2023)	Statin vs. non-user	Median (range): 21.1 (NA)	50/283 vs. 29/195	1.23 (0.75, 2.02)
Thorax, head and neck cancers					
Hepatitis rate	Boulet (2019)	Statin vs. non-user	Mean ± SD: statin user: 1.63 ± 1.93; non-user: 1.46 ± 1.88	20/4332 vs. 2/1386	3.21 (0.75, 13.75)
Transaminitis rate	Boulet (2019)	Statin vs. non-user	Mean ± SD: statin user: 1.63 ± 1.93; non-user: 1.46 ± 1.88	16/4332 vs. 1/1386	5.13 (0.68, 38.75)
Myositis/myalgia rate	Boulet (2019)	Statin vs. non-user	Mean ± SD: statin user: 1.63 ± 1.93; non-user: 1.46 ± 1.88	23/4332 vs. 4/1386	1.84 (0.64, 5.34)
Rhabdomyolysis rate	Boulet (2019)	Statin vs. non-user	Mean ± SD: statin user: 1.63 ± 1.93; non-user: 1.46 ± 1.88	1/4332 vs. 0/1386	0.64 (0.02, 19.09)
≥Grade 2 mucositis**	Sharifian (2024)	Statin vs. non-user	Median (range): statin user: 22 (NA); non-user: 17 (NA)	16/18 vs. 4/17	26.00 (4.09, 165.10) *
≥Grade 2 dermatitis	Sharifian (2024)	Statin vs. non-user	Median (range): statin user: 22 (NA); non-user: 17 (NA)	11/18 vs. 14/17	0.34 (0.07, 1.61)
≥Grade 2 dysphagia	Sharifian (2024)	Statin vs. non-user	Median (range): statin user: 22 (NA); non-user: 17 (NA)	7/18 vs. 6/17	1.17 (0.30, 4.61)
≥Grade 2 anaemia	Sharifian (2024)	Statin vs. non-user	Median (range): statin user: 22 (NA); non-user: 17 (NA)	2/18 vs. 2/17	0.94 (0.12, 7.52)
≥Grade 2 leukopenia	Sharifian (2024)	Statin vs. non-user	Median (range): statin user: 22 (NA); non-user: 17 (NA)	2/18 vs. 2/17	0.94 (0.12, 7.52)
≥Grade 2 thrombocytopenia	Sharifian (2024)	Statin vs. non-user	Median (range): statin user: 22 (NA); non-user: 17 (NA)	3/18 vs. 5/17	0.48 (0.09, 2.43)
Nasopharyngeal carcinoma					
Ischemic stroke rate	Lin (2024)	Statin vs. non-user	Median (range): 90 (NA)	273/2515 vs. 332/2507	0.80 (0.67, 0.95) *
Prostate cancer					
Rectal toxicity	Palumbo (2017)	Statin vs. non-user	Median (range): 26 (3, 60)	15/55 vs. 64/140	0.45 (0.23, 0.88) *
Acute genitourinary toxicity	Cadeddu (2020)	Statin vs. non-user	Median (range): 88 (1, 194)	NA/175 vs. NA/272 ^§^	1.20 (0.40, 1.00)
Chronic genitourinary toxicity	Cadeddu (2020)	Statin vs. non-user	Median (range): 88 (1, 194)	NA/175 vs. NA/272 ^§^	1.00 (0.60, 1.50)
Acute gastrointestinal toxicity	Cadeddu (2020)	Statin vs. non-user	Median (range): 88 (1, 194)	NA/175 vs. NA/272 ^§^	1.10 (0.70, 1.80)
Chronic gastrointestinal toxicity	Cadeddu (2020)	Statin vs. non-user	Median (range): 88 (1, 194)	NA/175 vs. NA/272 ^§^	1.50 (0.70, 2.90)

*NSCLC* non-small cell lung cancer, *IQR* interquartile range, *NA* not available. *SD* standard deviation, *CI* confidence interval

^§^The case number was not provided, but the odds ratio and 95% confidence interval were reported in the included study

*Significant difference

** Estimate for ≥ Grade 2 mucositis is derived from a single small RCT with high risk of bias and low certainty

## Discussion

This study found no significant difference in overall survival rates between statin users and non-users among patients with cancer receiving radiotherapy. However, progression-free and distant metastasis-free survival rates favoured statin users, albeit based on limited studies. The type of cancer influenced survival rates: statin use was associated with improved survival in oesophageal SCC, head and neck SCC, glioblastoma, and prostate cancer, but with poorer outcomes in NSCLC and brain metastases.

No correlation was found between the intensity of statin use and survival outcomes. Statin users had a higher incidence of major adverse cardiac events in NSCLC and ≥ grade 2 or greater mucositis in head and neck SCC, but a lower risk of ischemic stroke in nasopharyngeal carcinoma and rectal toxicity in prostate cancer. However, given that most of the included studies were observational, these findings demonstrate associations rather than causation.

This review found that the current evidence is limited in determining the impact of concurrent statin use on survival outcomes and radiotherapy-related adverse events. Moreover, many confounding factors were either not reported or could not be adequately controlled in regression analyses. The diverse biological characteristics, aggressiveness, and prognoses of different cancers may influence the effects of statins.

For example, statins inhibit the mevalonate pathway, leading to decreased cholesterol synthesis and the subsequent reduction of downstream isoprenoid intermediates, which can disrupt cancer cell signalling and tumour growth. However, the extent and nature of this disruption depend largely on the tumour’s genetic and molecular profile. NSCLC, for instance, frequently involves KRAS mutations and EGFR pathway alterations [11], which may interact differently with statin-induced modulation of signalling pathways compared to prostate cancer, where androgen receptor signalling and lipid metabolism are more prominent [12]. These molecular differences may partly explain why statins show anti-tumour effects in some cancers but not others. Additionally, factors such as the tumour microenvironment, immune modulation, and variations in statin lipophilicity and dosing could further contribute to the observed variability in outcomes [11, 12].

Patient characteristics (e.g., demographics, obesity, or smoking), cardiovascular conditions, comorbidities, and patient compliance also play significant roles [7, 17, 27, 29, 34]. Age is a crucial factor, as ischemic heart disease is more common in older patients [44] In some studies, statins users were significantly older [28] and had more comorbidities [7, 28]. In contrast, another study found that statin users had better disease characteristics, such as lower initial prostate-specific antigen levels [18], which may have impacted survival outcomes. Although some studies used propensity score matching or demographic analysis, they focused on cancer treatment effects, not statins [14, 19, 21, 31]. The duration of statin use is crucial, as cardiovascular benefits typically appear within a year, with significant effects after 3–4 years. Long-term benefits might result from cardiovascular effects rather than anti-cancer properties, and patients with certain cancers might not live long enough to experience these advantages [45].

Despite limited evidence, our study found that statin use is associated with higher overall survival in oesophageal SCC, head and neck SCC, glioblastoma, and prostate cancer. Previous studies suggest statins may benefit hormone-dependent cancers [10], such as prostate cancer after radical prostatectomy [46] or androgen deprivation therapy [47], and breast cancer undergoing various treatments [48, 49]. Statin users can benefit from the cholesterol-lowering effects, as cholesterol is a precursor to steroid hormones such as oestrogen and androgen, which play a role in developing various malignancies [10] However, statin use was associated with poorer outcomes in brain metastases patients, likely due to the worse prognosis of their cancer [33].

Furthermore, past studies have proposed a dose-response relationship, which is supported by two studies in our review: higher cumulative doses and intensities of statins are linked to lower oesophageal SCC-specific mortality during chemoradiotherapy [31] and higher overall survival in patients with NSCLC undergoing curative radiotherapy [26]. However, many included articles did not specify statin intensity or dosage, preventing a detailed analysis of this correlation. Consequently, only non-significant results were found regarding dose and overall survival rate.

Long-term statin therapy reduces major cardiovascular disease in patients with hypercholesterolemia [50, 51], but contrasting findings emerged in this review. Statin users with NSCLC, on the contrary, showed a significantly higher risk of major adverse cardiac events [34], whereas those with nasopharyngeal carcinoma had a lower rate of ischemic stroke [14]. Although statin users with head and neck SCC presented significantly more ≥ grade 2 mucositis, it was only reported by one study with a very wide 95%CI [43]. Other radiotherapy-related side effects associated with statin use were mainly non-significant or underreported in the studies included. Notably, this study found statin use was linked to reduced radiotherapy-related rectal toxicity in patients with prostate cancer, aligning with previous research on pravastatin’s potential to mitigate radiation proctitis [52].

This review systematically synthesised contemporary evidence using meta-analysis to investigate the impact of statin use on survival outcomes in patients with cancers undergoing radiotherapy, providing a comprehensive overview beyond individual studies. Meta-regression analysis examined the relationships between cancer type, statin intensity, and overall survival rates, aiming to identify relevant factors. However, the study is limited by confounding variables, including cancer type, stage, cardiovascular health, statin characteristics (type, dose, and duration), adherence, and follow-up period. Retrospective study designs may inadequately control for these factors, potentially introducing bias into the findings - heterogeneous reporting of adverse events restricted pooled meta-analysis. Furthermore, despite including two randomised clinical trials, both exhibited a high risk of bias, necessitating cautious interpretation of the study’s conclusions.

In the context of patients with cancers receiving radiotherapy, this study did not find a significant impact of statin use or intensity on overall survival outcomes. However, the analysis revealed an association between overall survival and specific types of cancer. Notably, statin users showed reduced rates of ischemic stroke in nasopharyngeal carcinoma and lower rectal toxicity in prostate cancer compared to non-users.

Based on these findings, several actionable clinical recommendations can be considered. For cancers such as prostate cancer, where evidence suggests potential benefits, clinicians might consider evaluating the use of statins as an adjunct to standard therapies, particularly in patients who have existing indications for lipid management or cardiovascular risk reduction. In head and neck squamous cell carcinoma (SCC), caution is warranted, and the decision to initiate or continue statins should involve a multidisciplinary assessment, considering factors such as tumour stage, patient comorbidities, and potential drug interactions.

In practice, for patients already receiving statins for cardiovascular indications, continuing therapy during radiotherapy appears reasonable, given the potential benefits and the importance of managing cardiovascular risk. In cases where statins are being considered solely for potential oncological benefit, personalised assessment of the risk-benefit ratio is essential, factoring in tumour type, molecular profile, and individual patient health status. Tailoring statin intensity and duration, starting with moderate doses and adjusting based on response and tolerability, may optimise outcomes while minimising adverse effects.

Furthermore, the variability in toxicity and cardiovascular outcomes across different tumour types underscores the importance of integrated cardio-oncology care. Future research, particularly prospective trials, is critical to identify specific patient subgroups, such as those with hormone-sensitive tumours like prostate cancer or specific molecular alterations, who may derive the most benefit from adjunctive statin therapy. Establishing evidence-based guidelines will ultimately facilitate more precise and effective utilisation of statins in oncology settings.

Future research is needed to explore molecular and mechanism-based biology to elucidate these findings further and enhance clinical understanding. Although randomised controlled trials are regarded as the best evidence to clarify the impact of statins on cancer radiotherapy, potential ethical and recruitment challenges are expected in patients with cancer. Alternatively, well-designed prospective cohort studies could offer valuable insights into statins’ influence, aiding causal inference. Our findings underscore the importance of refining inclusion criteria related to cardiovascular disease, cancer stage, statin characteristics (type, intensity, and duration), and follow-up period using propensity score matching to mitigate confounding bias.

## Conclusions

Clinically, while concurrent statin use did not significantly affect survival outcomes in cancer radiotherapy, clinicians may consider potential overall survival benefits in patients with oesophageal SCC, head and neck SCC, glioblastoma, and prostate cancer. Moreover, statin use may be associated with reduced rates of ischemic stroke in nasopharyngeal carcinoma and lower rectal toxicity in patients with prostate cancer. These findings highlight avenues for future research to explore and optimise statin therapy in the context of cancer treatment.

## Supplementary Information

Supplementary Material 1.

## Data Availability

The research data supporting the conclusions of this article are available upon request from the lead author.

## Abbreviations

OS: Overall survival
ORs: Odds ratios
95%CIs: 95% confidence intervals
SCC: Squamous cell carcinoma
NSCLC: Non-small cell lung cancer
PRISMA: Preferred Reporting Items for Systematic Reviews and Meta-Analyses
RoB 2: Risk of Bias Assessment Tool
ROBINS-I: Risk of Bias in Non-randomized Studies of Interventions tool
