# Supplementary material for: Impact of statin use on survival and adverse events in patients with cancer receiving radiotherapy: a systematic review and meta-analysis
Source: BMC Cancer. 2025 Oct 29;25:1666. doi: 10.1186/s12885-025-15038-3 (PMC12570484; doi:10.1186/s12885-025-15038-3)
Supplement: Supplementary file 1 — Supplementary Material 1. [file 12885_2025_15038_MOESM1_ESM.docx]

# Appendices

[Appendix 1. PRISMA 2020 Checklist 2](#_Toc174737393)

[**Appendix 2.** **Structured strategies for electronic database search** 4](#_Toc174737394)

[**Appendix 3.** **The risk-of-bias assessment for randomized controlled trials** 9](#_Toc174737395)

[Appendix 4. The risk-of-bias assessment for the cohort studies 10](#_Toc174737396)

Appendix 1. PRISMA 2020 Checklist

| **Section and Topic** | **Item #** | **Checklist item** | **Location where item is reported** |
| --- | --- | --- | --- |
| **TITLE** | | |  |
| Title | 1 | Identify the report as a systematic review. | Title page |
| **ABSTRACT** | | |  |
| Abstract | 2 | See the PRISMA 2020 for Abstracts checklist. | 1-2 |
| **INTRODUCTION** | | |  |
| Rationale | 3 | Describe the rationale for the review in the context of existing knowledge. | 2-4 |
| Objectives | 4 | Provide an explicit statement of the objective(s) or question(s) the review addresses. | 2-4 |
| **METHODS** | | |  |
| Eligibility criteria | 5 | Specify the inclusion and exclusion criteria for the review and how studies were grouped for the syntheses. | 4-7, Table 1 |
| Information sources | 6 | Specify all databases, registers, websites, organisations, reference lists and other sources searched or consulted to identify studies. Specify the date when each source was last searched or consulted. | 4-7 |
| Search strategy | 7 | Present the full search strategies for all databases, registers and websites, including any filters and limits used. | 4-5, Appendix 2 |
| Selection process | 8 | Specify the methods used to decide whether a study met the inclusion criteria of the review, including how many reviewers screened each record and each report retrieved, whether they worked independently, and if applicable, details of automation tools used in the process. | 4-7 |
| Data collection process | 9 | Specify the methods used to collect data from reports, including how many reviewers collected data from each report, whether they worked independently, any processes for obtaining or confirming data from study investigators, and if applicable, details of automation tools used in the process. | 4-7 |
| Data items | 10a | List and define all outcomes for which data were sought. Specify whether all results that were compatible with each outcome domain in each study were sought (e.g. for all measures, time points, analyses), and if not, the methods used to decide which results to collect. | 4-7 |
|  | 10b | List and define all other variables for which data were sought (e.g. participant and intervention characteristics, funding sources). Describe any assumptions made about any missing or unclear information. | 4-7 |
| Study risk of bias assessment | 11 | Specify the methods used to assess risk of bias in the included studies, including details of the tool(s) used, how many reviewers assessed each study and whether they worked independently, and if applicable, details of automation tools used in the process. | 6 |
| Effect measures | 12 | Specify for each outcome the effect measure(s) (e.g. risk ratio, mean difference) used in the synthesis or presentation of results. | 4-7 |
| Synthesis methods | 13a | Describe the processes used to decide which studies were eligible for each synthesis (e.g. tabulating the study intervention characteristics and comparing against the planned groups for each synthesis (item #5)). | 4-7 |
|  | 13b | Describe any methods required to prepare the data for presentation or synthesis, such as handling of missing summary statistics, or data conversions. | 4-7 |
|  | 13c | Describe any methods used to tabulate or visually display results of individual studies and syntheses. | 4-7 |
|  | 13d | Describe any methods used to synthesize results and provide a rationale for the choice(s). If meta-analysis was performed, describe the model(s), method(s) to identify the presence and extent of statistical heterogeneity, and software package(s) used. | 4-7 |
|  | 13e | Describe any methods used to explore possible causes of heterogeneity among study results (e.g. subgroup analysis, meta-regression). | 4-7 |
|  | 13f | Describe any sensitivity analyses conducted to assess robustness of the synthesized results. | 4-7 |
| Reporting bias assessment | 14 | Describe any methods used to assess risk of bias due to missing results in a synthesis (arising from reporting biases). | 4-7 |
| Certainty assessment | 15 | Describe any methods used to assess certainty (or confidence) in the body of evidence for an outcome. | 4-7 |
| **RESULTS** | | |  |
| Study selection | 16a | Describe the results of the search and selection process, from the number of records identified in the search to the number of studies included in the review, ideally using a flow diagram. | 7, Figure 1 |
|  | 16b | Cite studies that might appear to meet the inclusion criteria, but which were excluded, and explain why they were excluded. | 7, Figure 1 |
| Study characteristics | 17 | Cite each included study and present its characteristics. | 7-8, Table 2 |
| Risk of bias in studies | 18 | Present assessments of risk of bias for each included study. | 8, Appendix 3 and 4 |
| Results of individual studies | 19 | For all outcomes, present, for each study: (a) summary statistics for each group (where appropriate) and (b) an effect estimate and its precision (e.g. confidence/credible interval), ideally using structured tables or plots. | 7-10, Table 3-6 |
| Results of syntheses | 20a | For each synthesis, briefly summarise the characteristics and risk of bias among contributing studies. | 7-10 |
|  | 20b | Present results of all statistical syntheses conducted. If meta-analysis was done, present for each the summary estimate and its precision (e.g. confidence/credible interval) and measures of statistical heterogeneity. If comparing groups, describe the direction of the effect. | 7-10, Table 3-6 |
|  | 20c | Present results of all investigations of possible causes of heterogeneity among study results. | 7-10 |
|  | 20d | Present results of all sensitivity analyses conducted to assess the robustness of the synthesized results. | 7-10 |
| Reporting biases | 21 | Present assessments of risk of bias due to missing results (arising from reporting biases) for each synthesis assessed. | 7-10 |
| Certainty of evidence | 22 | Present assessments of certainty (or confidence) in the body of evidence for each outcome assessed. | 7-10 |
| **DISCUSSION** | | |  |
| Discussion | 23a | Provide a general interpretation of the results in the context of other evidence. | 10-11 |
|  | 23b | Discuss any limitations of the evidence included in the review. | 10-14 |
|  | 23c | Discuss any limitations of the review processes used. | 10-14 |
|  | 23d | Discuss implications of the results for practice, policy, and future research. | 10-14 |
| **OTHER INFORMATION** | | |  |
| Registration and protocol | 24a | Provide registration information for the review, including register name and registration number, or state that the review was not registered. | 4 |
|  | 24b | Indicate where the review protocol can be accessed, or state that a protocol was not prepared. | 4 |
|  | 24c | Describe and explain any amendments to information provided at registration or in the protocol. | 4 |
| Support | 25 | Describe sources of financial or non-financial support for the review, and the role of the funders or sponsors in the review. | 14-15 |
| Competing interests | 26 | Declare any competing interests of review authors. | 14 |
| Availability of data, code and other materials | 27 | Report which of the following are publicly available and where they can be found: template data collection forms; data extracted from included studies; data used for all analyses; analytic code; any other materials used in the review. | Not applicable |

*From:*  Page MJ, McKenzie JE, Bossuyt PM, Boutron I, Hoffmann TC, Mulrow CD, et al. The PRISMA 2020 statement: an updated guideline for reporting systematic reviews. BMJ 2021;372:n71. doi: 10.1136/bmj.n71

**Appendix 2. Structured strategies for electronic database search**

**MEDLINE**

1 exp Radiotherapy/

2 exp Radiation/

3 exp Chemoradiotherapy/

4 exp Radiotherapy, Computer-Assisted/

5 Stereotactic body radiation therapy.mp.

6 Stereotactic radiosurgery.mp. or Radiosurgery/

7 Definitive Radiation Therapy.mp.

8 Thoracic Irradiation.mp.

9 Total body irradiation.mp. or Whole-Body Irradiation/

10 Radiotherap*.mp.

11 Radiother*.mp.

12 Radiat*.mp.

13 Irradiat*.mp.

14 (Radiochemo* or Chemoradio*).mp. [mp=title, book title, abstract, original title, name of substance word, subject heading word, floating sub-heading word, keyword heading word, organism supplementary concept word, protocol supplementary concept word, rare disease supplementary concept word, unique identifier, synonyms, population supplementary concept word, anatomy supplementary concept word]

15 Radiation therap*.mp.

16 1 or 2 or 3 or 4 or 5 or 6 or 7 or 8 or 9 or 10 or 11 or 12 or 13 or 14 or 15

17 exp Anticholesteremic Agents/

18 exp Hydroxymethylglutaryl CoA Reductases/

19 (hydroxymethylglutaryl* adj5 inhibitor*).tw.

20 (hmg-coa* adj5 statin*).tw.

21 (hmg-coa* adj5 inhibit*).tw.

22 3-hydroxy-3-methylpentanedioic acid.tw.

23 beta-hydroxy-beta-methylglutarate.tw.

24 3-hydroxy-3-methylglutaric acid.tw.

25 Statin*.tw.

26 (altoc?r or altoprev or artein or atorvastatin).tw.

27 (baycol or bristacol or "bay w 6228" or "bay w6228").tw.

28 (canef or cerivastatin or certa or compactin or cranoc or crestor or ci-981 or ci981 or cs-500 or cs500 or cs-514 or cs514).tw.

29 (dalvastatin or denan).tw.

30 (elisor or epatostantin or eptastatin* or epistatin or fluindostatin or fluvastatin or gerosim or itavastatin).tw.

31 (lescol or leucol or lipemol or lipitor or lipibec or liplat or lipex or lipobay or lipovas or lipostat or livalo or loc?ol or lodales or lovacol or lovastatin or l-654969 or l-644128 or l644128).tw.

32 (mevastatin or mevastin or mevinolin or mona?olin or methylcompactin or mk-803 or mk803 or mk-0803 or mk0803 or msd-803 or mevacor or mk-733 or mk733 or meglutol or mevalotin or mevinacor or medostatin or ml-236b or ml236b or medipo).tw.

33 (nk-104 or nk104 or nks-104 or nks104 or nisvastatin or neolipid).tw.

34 (pravastatin or prareduct or pravachol or pravacol or pravasin* or pitavastatin or pitava or pravachol).tw.

35 (rms-431 or rms431 or ribar or rivastatin or rosuvastatin or RG-12561).tw.

36 (sanaprav or selektine or simvastatin or sinvacor or s?nvinolin or sortis or sq-31000 or sq31000 sq-31,000 or sq31,000 or sri-62320 or sri62320 or s-4522 or s4522).tw.

37 (tahor or torvast).tw.

38 (vast?n or xu-62320 or xu62320 or ym-548 or ym548 or zarator or zenas or zocor? or zd-4522 or zd4522).tw.

39 atorvastatin/

40 Fluvastatin.mp. or fluindostatin/

41 Lovastatin.mp. or mevinolin/

42 pitavastatin/

43 pravastatin/

44 rosuvastatin/

45 simvastatin/

46 cerivastatin/

47 zocor.mp.

48 17 or 18 or 19 or 20 or 21 or 22 or 23 or 24 or 25 or 26 or 27 or 28 or 29 or 30 or 31 or 32 or 33 or 34 or 35 or 36 or 37 or 38 or 39 or 40 or 41 or 42 or 43 or 44 or 45 or 46 or 47

49 16 and 48

50 limit 49 to (english language and humans and yr="2000 -Current" and "all adult (19 plus years)")

**EMBASE**

1 exp Radiotherapy/

2 exp Radiation/

3 exp Chemoradiotherapy/

4 exp Radiotherapy, Computer-Assisted/

5 Stereotactic body radiation therapy.mp.

6 Stereotactic radiosurgery.mp. or Radiosurgery/

7 Definitive Radiation Therapy.mp.

8 Thoracic Irradiation.mp.

9 Total body irradiation.mp. or Whole-Body Irradiation/

10 Radiotherap*.mp.

11 Radiother*.mp.

12 Radiat*.mp.

13 Irradiat*.mp.

14 (Radiochemo* or Chemoradio*).mp. [mp=title, abstract, heading word, drug trade name, original title, device manufacturer, drug manufacturer, device trade name, keyword heading word, floating subheading word, candidate term word]

15 Radiation therap*.mp.

16 1 or 2 or 3 or 4 or 5 or 6 or 7 or 8 or 9 or 10 or 11 or 12 or 13 or 14 or 15

17 exp Anticholesteremic Agents/

18 exp Hydroxymethylglutaryl CoA Reductases/

19 (hydroxymethylglutaryl* adj5 inhibitor*).tw.

20 (hmg-coa* adj5 statin*).tw.

21 (hmg-coa* adj5 inhibit*).tw.

22 3-hydroxy-3-methylpentanedioic acid.tw.

23 beta-hydroxy-beta-methylglutarate.tw.

24 3-hydroxy-3-methylglutaric acid.tw.

25 Statin*.tw.

26 (altoc?r or altoprev or artein or atorvastatin).tw.

27 (baycol or bristacol or "bay w 6228" or "bay w6228").tw.

28 (canef or cerivastatin or certa or compactin or cranoc or crestor or ci-981 or ci981 or cs-500 or cs500 or cs-514 or cs514).tw.

29 (dalvastatin or denan).tw.

30 (elisor or epatostantin or eptastatin* or epistatin or fluindostatin or fluvastatin or gerosim or itavastatin).tw.

31 (lescol or leucol or lipemol or lipitor or lipibec or liplat or lipex or lipobay or lipovas or lipostat or livalo or loc?ol or lodales or lovacol or lovastatin or l-654969 or l-644128 or l644128).tw.

32 (mevastatin or mevastin or mevinolin or mona?olin or methylcompactin or mk-803 or mk803 or mk-0803 or mk0803 or msd-803 or mevacor or mk-733 or mk733 or meglutol or mevalotin or mevinacor or medostatin or ml-236b or ml236b or medipo).tw.

33 (nk-104 or nk104 or nks-104 or nks104 or nisvastatin or neolipid).tw.

34 (pravastatin or prareduct or pravachol or pravacol or pravasin* or pitavastatin or pitava or pravachol).tw.

35 (rms-431 or rms431 or ribar or rivastatin or rosuvastatin or RG-12561).tw.

36 (sanaprav or selektine or simvastatin or sinvacor or s?nvinolin or sortis or sq-31000 or sq31000 sq-31,000 or sq31,000 or sri-62320 or sri62320 or s-4522 or s4522).tw.

37 (tahor or torvast).tw.

38 (vast?n or xu-62320 or xu62320 or ym-548 or ym548 or zarator or zenas or zocor? or zd-4522 or zd4522).tw.

39 atorvastatin/

40 Fluvastatin.mp. or fluindostatin/

41 Lovastatin.mp. or mevinolin/

42 pitavastatin/

43 pravastatin/

44 rosuvastatin/

45 simvastatin/

46 cerivastatin/

47 zocor.mp.

48 17 or 18 or 19 or 20 or 21 or 22 or 23 or 24 or 25 or 26 or 27 or 28 or 29 or 30 or 31 or 32 or 33 or 34 or 35 or 36 or 37 or 38 or 39 or 40 or 41 or 42 or 43 or 44 or 45 or 46 or 47

49 16 and 48

50 limit 49 to (human and english language and yr="2000 -Current" and adult <18 to 64 years>)

**Web of Science**

(Radiotherapy or Radiation or Chemoradiotherapy or Stereotactic body radiation therapy or Stereotactic radiosurgery or Radiosurgery or Definitive Radiation Therapy or Thoracic Irradiation or Total Body Irradiation or Whole-Body Irradiation or Radiotherapy-Computer-Assisted or Radiotherap* or Radiother* or Radiat* or Irradiat* or Radiochemo* or Chemoradio* or Radiation therap*) and (Anticholesteremic Agents or Hydroxymethylglutaryl CoA Reductases or 3-hydroxy-3-methylpentanedioic acid or beta-hydroxy-beta-methylglutarate or 3-hydroxy-3-methylglutaric acid or Statin* or altoc?r or altoprev or artein or atorvastatin or baycol or bristacol or "bay w 6228" or "bay w6228" or canef or cerivastatin or certa or compactin or cranoc or crestor or ci-981 or ci981 or cs-500 or cs500 or cs-514 or cs514 or dalvastatin or denan or elisor or epatostantin or eptastatin* or epistatin or fluindostatin or fluvastatin or gerosim or itavastatin or lescol or leucol or lipemol or lipitor or lipibec or liplat or lipex or lipobay or lipovas or lipostat or livalo or loc?ol or lodales or lovacol or lovastatin or l-654969 or l-644128 or l644128 or mevastatin or mevastin or mevinolin or mona?olin or methylcompactin or mk-803 or mk803 or mk-0803 or mk0803 or msd-803 or mevacor or mk-733 or mk733 or meglutol or mevalotin or mevinacor or medostatin or ml-236b or ml236b or medipo or nk-104 or nk104 or nks-104 or nks104 or nisvastatin or neolipid or pravastatin or prareduct or pravachol or pravacol or pravasin* or pitavastatin or pitava or Pravachol or rms-431 or rms431 or ribar or rivastatin or rosuvastatin or RG-12561 or sanaprav or selektine or simvastatin or sinvacor or s?nvinolin or sortis or sq-31000 or sq31000 sq-31,000 or sq31,000 or sri-62320 or sri62320 or s-4522 or s4522 or tahor or torvast or vast?n or xu-62320 or xu62320 or ym-548 or ym548 or zarator or zenas or zocor? or zd-4522 or zd4522)

**Scopus**

( radiotherapy OR radiation OR chemoradiotherapy OR stereotactic AND body AND radiation AND therapy OR stereotactic AND radiosurgery OR radiosurgery OR definitive AND radiation AND therapy OR thoracic AND irradiation OR total AND body AND irradiation OR whole-body AND irradiation OR radiotherapy-computer-assisted OR radiotherap* OR radiother* OR radiat* OR irradiat* OR radiochemo* OR chemoradio* OR radiation AND therap* ) AND statin*

**PubMed**

((radiotherapy) OR (radiation) OR (chemoradiotherapy) OR (radiosurgery) OR (irradiation) OR (radiotherapy-computer-assisted)) AND ((statin) OR (atorvastatin) OR (dalvastatin) OR (fluindostatin) OR (fluvastatin) OR (itavastatin) OR (lipitor) OR (lovastatin) OR (mevastatin) OR (nisvastatin) OR (pravastatin) OR (pitavastatin) OR (rivastatin) OR (rosuvastatin) OR (simvastatin))

**Appendix 3. The risk-of-bias assessment for randomized controlled trials**

| **Author, year** | **Bias arising from the randomisation process** | **Bias due to deviations from intended intervention** | **Bias due to missing outcome data** | **Bias in the measurement of the outcome** | **Bias in the selection of the reported result** | **Overall** |
| --- | --- | --- | --- | --- | --- | --- |
| El-Hamamsy, 2016 | High risk | Low risk | High risk | High risk | Low risk | High risk |
| Sharifian, 2024 | Some concerns | Low risk | High risk | Low risk | Low risk | High risk |

(Note) The risk-of-bias tool for randomized trials (RoB 2) was applied. Background color caption: green: low risk of bias; yellow: some concerns; and red: high risk of bias.

Appendix 4. The risk-of-bias assessment for the cohort studies

| **Author, year** | **Bias due to confounding** | **Bias due to the selection of participants** | **Bias in the classification of interventions** | **Bias due to deviations from intended interventions** | **Bias due to missing data** | **Bias in the measurement of outcomes** | **Bias in the selection of the reported result** | **Overall** |
| --- | --- | --- | --- | --- | --- | --- | --- | --- |
| Moyad, 2006 | Moderate | Low | Moderate | Low | Low | Low | Serious | Serious |
| Soto, 2009 | Moderate | Low | Low | Low | Low | Moderate | Low | Moderate |
| Gutt, 2010 | Moderate | Low | Low | Low | Moderate | Low | Low | Moderate |
| Kollmeier, 2011 | Moderate | Low | Low | Low | Low | Low | Serious | Serious |
| Alizadeh, 2012 | Low | Low | Low | Low | Low | Low | Serious | Serious |
| Wedlake, 2012 | Moderate | Low | Low | Low | Moderate | Moderate | Serious | Serious |
| Chao, 2013 | Moderate | Low | Low | Low | Low | Moderate | Serious | Serious |
| Caon, 2014 | Moderate | Low | Low | Low | Moderate | Moderate | Low | Moderate |
| Cuaron, 2015 | Moderate | Low | Low | Low | Low | Low | Serious | Serious |
| Oh, 2015 | Moderate | Low | Low | Low | Low | Low | Low | Moderate |
| Liu, 2017 | Moderate | Low | Low | Low | Low | Low | Low | Moderate |
| Palumbo, 2017 | Moderate | Low | Moderate | Low | Low | Low | Low | Moderate |
| Boulet, 2019 | Moderate | Low | Low | Low | Low | Low | Serious | Serious |
| Cadeddu, 2020 | Moderate | Low | Moderate | Low | Low | Low | Low | Moderate |
| Altwairgi, 2021 | Moderate | Moderate | Low | Low | Low | Serious | Serious | Serious |
| Atkins, 2021 | Moderate | Low | Low | Low | Low | Low | Low | Moderate |
| Chen, 2023 | Moderate | Low | Low | Low | Low | Moderate | Low | Moderate |
| Walls, 2023 | Moderate | Low | Low | Low | Moderate | Low | Serious | Serious |
| Lin, 2024 | Moderate | Low | Low | Low | Low | Moderate | Low | Moderate |

(Note) The risk of bias in non-randomized studies of interventions (ROBINS-I) tool was applied. Background color caption: green; low risk of bias (the study is comparable to a well-performed randomized trial concerning this domain); yellow: moderate risk of bias (the study is sound for a non-randomized study concerning this domain but cannot be considered comparable to a well-performed randomized trial); orange: serious risk of bias (the study has some important problems); and red critical risk of bias (the study is too problematic to provide any useful evidence on the effects of intervention).
